# State Estimation for a Class of Non-Uniform Sampling Systems with Missing Measurements

**DOI:** 10.3390/s16081155

**Published:** 2016-07-23

**Authors:** Honglei Lin, Shuli Sun

**Affiliations:** School of Electronics Engineering, Heilongjiang University, Harbin 150080, China; linhonglei0810@163.com

**Keywords:** modeling, non-uniform sampling, missing measurement, non-augmented estimator, innovation analysis approach

## Abstract

This paper is concerned with the state estimation problem for a class of non-uniform sampling systems with missing measurements where the state is updated uniformly and the measurements are sampled randomly. A new state model is developed to depict the dynamics at the measurement sampling points within a state update period. A non-augmented state estimator dependent on the missing rate is presented by applying an innovation analysis approach. It can provide the state estimates at the state update points and at the measurement sampling points within a state update period. Compared with the augmented method, the proposed algorithm can reduce the computational burden with the increase of the number of measurement samples within a state update period. It can deal with the optimal estimation problem for single and multi-sensor systems in a unified way. To improve the reliability, a distributed suboptimal fusion estimator at the state update points is also given for multi-sensor systems by using the covariance intersection fusion algorithm. The simulation research verifies the effectiveness of the proposed algorithms.

## 1. Introduction

Recently, the estimation problems for multi-rate non-uniform sampling systems have attracted much attention due to wide applications in parameter identification [1], industrial detection [2], target tracking and signal processing [3,4,5,6,7,8]. Differently from the single-rate uniform sampling systems, the design on multi-rate non-uniform sampling systems is more complex and challenging.

For multi-rate non-uniform sampling systems, the multi-sensor information fusion filters have been presented based on the data block method in [3,4,5] where the effect of the system noise on modeling is ignored, which brings the errors in modeling. By considering the system noise in modeling, an optimal estimator in the linear minimum variance sense is presented in [6]. The modeling methods in [4,5,6] are, respectively, adopted to obtain the distributed fusion filters by the weighting sums of the local filters in [7,8]. In the literature above, the state models are all from weighted average of states in a data block. When there are multiple measurement samples within a state update period, a state estimator is designed by the measurement augmentation method in [9], which brings expensive computational cost. The discretization of continuous systems is adopted in the multi-rate processing in [10,11].

The distributed fusion filters are designed for multi-rate multi-sensor systems in [12,13]. In [12], an estimator is proposed for systems with uncertain observations. In [13], a “dummy” measurement is used to transform multi-rate into single-rate for each sensor and the packet dropouts are considered when the measurements are transmitted by networks. For the multi-sensor networked systems with packet dropouts, a two-stage distributed fusion estimation algorithm is proposed by using a multi-rate scheme to reduce communication cost in [14], where sensors collect measurements from their neighbors to generate their own local estimates, and then local estimates are collected to form a fused estimate. The multi-rate $H_{\infty }$ filtering problem for the norm-bounded uncertain systems with packet dropouts is investigated by the state augmentation method in [15]. The multi-rate filter in the least mean square sense is designed in [16] and can obtain more accurate estimate than the $H_{\infty }$ filter in [15]. In these studies, the sampling periods of individual sensors are uniform and integer times of the state update period though different sensors have different sampling rates. The two-sensor multi-rate fusion estimation problem for wireless sensor networks is investigated in [17]. Then, the results in [17] are improved and extended in [18]. However, the sampling period of each sensor is still positive integer times of the state update period. A novel method that is named direct estimation of sampling time in solving asynchronous track-to-track fusion problem in [19] is used to predict the pseudo-synchronized state estimates of all the sensors that possess their own sampling rates for the start of the next fusion period. A suboptimal hierarchical fusion estimator is designed in [20] for a clustered sensor network by using covariance intersection fusion algorithm, where local estimators and the fusion center are allowed to be asynchronous. Information fusion estimation problem in multi-sensor networked systems is also explored in [21,22,23,24] where packet dropouts, random delays and missing measurements are considered. However, the multi-rate asynchronous estimation problems are not taken into account.

In practice, not only different sensors can have different sampling rates but also the same sensor can also have asynchronous sampling rates. Moreover, the state estimators need to be obtained in real time for many practical applications. Early work has been done in [25] where the real time estimation can be obtained not only at the state update points but also at the measurement sampling points. However, the single sensor optimal estimation problem is only considered in [25] in the case that the sensor randomly samples the measurement once at most in a state update period. Motivated by the above discussion, we carry out our work in this paper. For systems with the uniformly updated state and randomly sampled measurements, where missing measurements are also considered, a new state space model is developed to depict the dynamics at the measurement sampling points within a state update period by weighting the endpoint states of a state update period. Differently from [25] where sensor randomly samples one measurement at most in a state update period, sensor randomly samples one or multiple measurements or nothing in a state update period in this paper. A non-augmented recursive estimator at the measurement sampling points and at the state update points is designed by applying projection theory. Compared with the augmented method [9], the proposed algorithm can reduce the computational burden and provide the estimates at the measurement sampling points within a state update period. At last, the optimal fusion and distributed suboptimal fusion estimators are given to deal with the fusion estimation problems for multi-sensor systems.

## 2. Problem Formulation

Consider the following non-uniform sampling discrete time-invariant linear stochastic system with missing measurements.

(1) x(k+1)=Φx(k)+Γw(k)

(2) $$y(k_{j})=\xi (k_{j})Hx(k_{j})+v(k_{j})$$

where $x(k)\in R^{p}$ is the system state at the moment kT, the Italic T is the state sampling period, and $y(k_{j})\in R^{q}$ is the measurement of the sensor at the moment $k_{j}$. $x(k_{j})$ is the state measured by $y(k_{j})$. $w(k)\in R^{r}$ and $v(k_{j})\in R^{q}$ are the system noise and measurement noise. Φ, Γ and H are constant matrices with suitable dimensions. $k_{j}$ is the sampling time of the jth measurement. The variable $\xi (k_{j})$ is a Bernoulli distributed stochastic variable that takes values on 1 and 0 with the probability $\text{Prob}\{\xi (k_{j})=1\}=\bar{\gamma }$, $\bar{\gamma }\in [0,1]$. $\xi (k_{j})=1$ means that the measurement is received, while $\xi (k_{j})=0$ means that the measurement is missing. In the whole text, E denotes the mathematical expectation and the superscript Roman *T* denotes the transpose. We easily obtain the results:

(3) $$E\{\xi (k_{j})\}=\bar{\gamma },\;E\{{\left(\xi (k_{j})-\bar{\gamma }\right)}^{2}\}=\bar{\gamma }(1-\bar{\gamma }),\;E\{\xi (k_{j})(1-\xi (k_{j}))\}=0,\;E\{\xi (k_{i})\xi (l_{j})\}={\bar{\gamma }}^{2}(k_{i}\neq l_{j})$$

For brevity of notations, only linear time-invariant systems are considered. However, the results derived later can be easily extended to linear time-varying systems. We adopt the following standard assumptions.

**Assumption 1.**  w(k) *and* $v(k_{j})$ *are uncorrelated white noises with zero means and covariance matrices* $Q_{w}$ *and* $Q_{v}$.

**Assumption 2.**  *The initial state* x(0) *is uncorrelated with* w(k)*,* $v(k_{j})$ *and* $\xi (k_{j})$*, and satisfies*

(4) $$E\left\{x(0)\right\}=\mu_{0},\;E\left\{(x(0)-\mu_{0}){\left(x(0)-\mu_{0}\right)}^{T}\right\}=P_{0}$$

**Figure 1:**
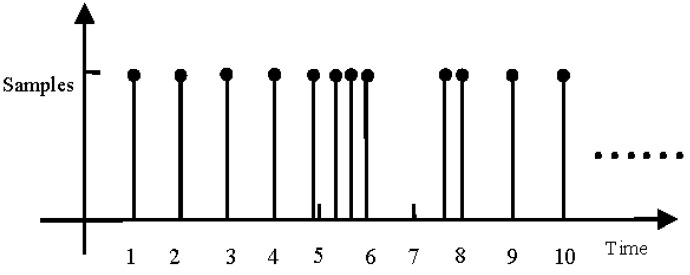
Illustration of non-uniform sampling.

Assuming that the sampling time of the sensor is known, we consider a class of non-uniform sampling discrete-time systems with missing measurements where the state updates uniformly and the sensor samples randomly in this paper. An example of sampling time versus sensor map is depicted in Figure 1. It can be seen from Figure 1 that there are two cases when the sensor samples the measurement data. Case 1 is that there are the measurement samples in a state update period ((k−1)T,kT], for example, only one measurement sample at the time instants *T*, 2*T*, 3*T*, 4*T*, 9*T*, 10*T* and within the interval (4T,5T] with the sampling time instants $k_{1}=1$, $k_{2}=2$, $k_{3}=3$, $k_{4}=4$, $k_{11}=9$, $k_{12}=10$, $k_{5}=4.85$, two samples in (7T,8T] with the sampling time instants$k_{9}=7.6$, $k_{10}=8$, and three samples within (5T,6T] with the sampling time instants $k_{6}=5.35$, $k_{7}=5.65$, $k_{8}=6$. Case 2 is that there are no samples within the state update period ((k−1)T,kT], for example, not any measurement sample within (6T,7T].

Our aim is to find the state filters at state update points and measurement sampling points based on the received measurements.

## 3. System Modeling

Assume that the sensor starts to sample the measurement at the initial moment. When the sensor samples a measurement at the state update point, it is natural to take the system state at the state update point as the state at the measurement sampling point. When the sensor samples a measurement between the two state update points, we adopt the method in [9] where the weighted value of the two adjacent states is taken as the state at the measurement sampling point.

In order to facilitate the algorithm, let $N_{k}$ is the number of measurement samples within a state update interval ((k−1)T,kT], and $S_{k}=\sum_{l=1}^{k}N_{l}$ is the total number of measurement samples before the moment kT. The i ($1\leq i\leq N_{k}$) denotes the ith sample within the interval ((k−1)T,kT]. $k=\left\lceil k_{S_{k-1}+i}/T\right\rceil$ where ⌈∗⌉ denotes the minimal integer not less than ∗. Let $\alpha_{i}^{\left(k\right)}=k-k_{S_{k-1}+i}/T$, and then we have $k_{S_{k-1}+i}=k-\alpha_{i}^{\left(k\right)}$. It is easily known that $0\leq \alpha_{i}^{\left(k\right)}<1$. To simplify the expression, the sampling period T will be omitted under no confusion in the subsequent text.

The state at the measurement sampling point within the interval (k−1,k] is given by

(5) $$x(k-\alpha_{i}^{\left(k\right)})=(1-\alpha_{i}^{\left(k\right)})x(k)+\alpha_{i}^{\left(k\right)}x(k-1)$$

Accordingly, the measurement equation is expressed as

(6) $$y(k-\alpha_{i}^{\left(k\right)})=\xi (k-\alpha_{i}^{\left(k\right)})Hx(k-\alpha_{i}^{\left(k\right)})+v(k-\alpha_{i}^{\left(k\right)})$$

Especially, when the measurement is sampled at the state update point, we have $\alpha_{i}^{\left(k\right)}=0$. Then the state at the measurement sampling point can be reduced as x(k). The measurement equation can be reduced as y(k)=ξ(k)Hx(k)+v(k).

Assuming i and i−1 are two adjacent sampling points in (k−1,k]. From Equation (5), we have

(7) $$x(k)=\frac{1}{1-\alpha_{i-1}^{\left(k\right)}}x(k-\alpha_{i-1}^{\left(k\right)})-\frac{\alpha_{i-1}^{\left(k\right)}}{1-\alpha_{i-1}^{\left(k\right)}}x(k-1)$$

Substituting Equation (7) into Equation (5) yields

(8) $$x(k-\alpha_{i}^{\left(k\right)})=\frac{\left(1-\alpha_{i}^{\left(k\right)}\right)}{\left(1-\alpha_{i-1}^{\left(k\right)}\right)}x(k-\alpha_{i-1}^{\left(k\right)})+\frac{\left(\alpha_{i}^{\left(k\right)}-\alpha_{i-1}^{\left(k\right)}\right)}{\left(1-\alpha_{i-1}^{\left(k\right)}\right)}x(k-1)$$

Next, we construct the state space model at the measurement sampling points within a state update period. The following Theorem 1 gives the result.

**Theorem 1.**  *Under Assumptions 1 and 2, the state space model at the measurement sampling points within the interval (k−1,k] can be set up as follows:*

(9) $$x(k-\alpha_{i}^{\left(k\right)})=\Phi_{i}^{\left(k\right)}x(k-1)+\Gamma_{i}^{\left(k\right)}w(k-1)$$

(10) $$y(k-\alpha_{i}^{\left(k\right)})=\xi (k-\alpha_{i}^{\left(k\right)})Hx(k-\alpha_{i}^{\left(k\right)})+v(k-\alpha_{i}^{\left(k\right)})$$

*where the coefficient matrices* $\Phi_{i}^{\left(k\right)}$ *and* $\Gamma_{i}^{\left(k\right)}$ *are defined by*

(11) $$\Phi_{i}^{\left(k\right)}=\prod_{m=0}^{i-1}\beta_{i-m}^{\left(k\right)}\Phi +\sum_{l=0}^{i-1}\left(\prod_{m=0}^{l-1}\beta_{i-m}^{\left(k\right)}\right)(1-\beta_{i-l}^{\left(k\right)})I,\;\Gamma_{i}^{\left(k\right)}=\prod_{m=0}^{i-1}\beta_{i-m}^{\left(k\right)}\Gamma$$

*with $\beta_{i}^{\left(k\right)}=(1-\alpha_{i}^{\left(k\right)})/(1-\alpha_{i-1}^{\left(k\right)}),(i>1)$, $\beta_{1}^{\left(k\right)}=1-\alpha_{1}^{\left(k\right)}$ and $\prod_{m=0}^{-1}\beta_{i-m}^{\left(k\right)}=1$.*

**Proof.**  From Equation (8), we have

(12) $$x(k-\alpha_{i}^{\left(k\right)})=\beta_{i}^{\left(k\right)}x(k-\alpha_{i-1}^{\left(k\right)})+(1-\beta_{i}^{\left(k\right)})x(k-1)$$

From Equation (5), we obtain

(13) $$x(k-\alpha_{1}^{\left(k\right)})=\beta_{1}^{\left(k\right)}x(k)+(1-\beta_{1}^{\left(k\right)})x(k-1)$$

By iterating Equation (12) and using Equation (13), we have

(14) $$\begin{array}{l} x(k-\alpha_{i}^{\left(k\right)})=\beta_{i}^{\left(k\right)}x(k-\alpha_{i-1}^{\left(k\right)})+(1-\beta_{i}^{\left(k\right)})x(k-1)\\ =\beta_{i}^{\left(k\right)}\beta_{i-1}^{\left(k\right)}x(k-\alpha_{i-2}^{\left(k\right)})+\beta_{i}^{\left(k\right)}(1-\beta_{i-1}^{\left(k\right)})x(k-1)+(1-\beta_{i}^{\left(k\right)})x(k-1)\\ =\cdots =\beta_{i}^{\left(k\right)}\beta_{i-1}^{\left(k\right)}\cdots \beta_{1}^{\left(k\right)}x(k)+\beta_{i}^{\left(k\right)}\beta_{i-1}^{\left(k\right)}\cdots \beta_{2}^{\left(k\right)}(1-\beta_{1}^{\left(k\right)})x(k-1)+\cdots +\beta_{i}^{\left(k\right)}(1-\beta_{i-1}^{\left(k\right)})x(k-1)+(1-\beta_{i}^{\left(k\right)})x(k-1)\\ =\prod_{m=0}^{i-1}\beta_{i-m}^{\left(k\right)}\left(\Phi x(k-1)+\Gamma w(k-1)\right)+\sum_{l=0}^{i-1}\left(\prod_{m=0}^{l-1}\beta_{i-m}^{\left(k\right)}\right)(1-\beta_{i-l}^{\left(k\right)})x(k-1)\\ =\Phi_{i}^{\left(k\right)}x(k-1)+\Gamma_{i}^{\left(k\right)}w(k-1) \end{array}$$

where $\Phi_{i}^{\left(k\right)}$ and $\Gamma_{i}^{\left(k\right)}$ are defined by Equation (11). The proof is completed.

**Remark 1.**  *The model proposed in Theorem 1 establishes the relationship between the state at the measurement sampling points within the interval (k−1,k]. It includes the single rate uniform sampling system as the special case, i.e., $N_{k}=1$ and $\alpha_{1}^{\left(k\right)}=0$. Thus, it generalizes the standard single rate discrete-time state space model*.

To design the filtering algorithm, we first give a lemma to be used in the subsequent sections.

**Lemma 1.**  *For system Equation (9) under Assumptions 1 and 2, the state second-order moment matrix $q_{x}(k-\alpha_{i}^{\left(k\right)})=E\left\{x(k-\alpha_{i}^{\left(k\right)})x^{T}(k-\alpha_{i}^{\left(k\right)})\right\}$ is computed by*

(15) $$q_{x}(k-\alpha_{i}^{\left(k\right)})=\Phi_{i}^{k}q_{x}(t-1){\left(\Phi_{i}^{k}\right)}^{T}+\Gamma_{i}^{k}Q_{w}{\left(\Gamma_{i}^{k}\right)}^{T}$$

*The initial value is* $q_{x}(0)=\mu_{0}\mu_{0}^{T}+P_{0}$.

**Proof.**  Substituting Equation (9) into $q_{x}(k-\alpha_{i}^{\left(k\right)})=E\left\{x(k-\alpha_{i}^{\left(k\right)})x^{T}(k-\alpha_{i}^{\left(k\right)})\right\}$, we easily obtain Equation (15).

## 4. Optimal State Estimator

In this section, we will present our estimation algorithm at the state update points and measurement sampling points based on the developed model in Theorem 1.

### 4.1. Estimator Design

**Theorem 2.**  *For system Equations (9) and (10) under Assumptions 1 and 2, the optimal estimators at the state update point and measurement sampling points in the interval (k−1,k]($N_{k}\neq 0$) are computed by*

(16) $$\hat{x}(k-\alpha_{i}^{\left(k\right)}|k-\alpha_{i-r}^{\left(k\right)})=\Phi_{i}^{\left(k\right)}\hat{x}(k-1|k-\alpha_{i-r}^{\left(k\right)})+\Gamma_{i}^{\left(k\right)}\hat{w}(k-1|k-\alpha_{i-r}^{\left(k\right)}),r=0,1$$

*where it is a filter for r=0 or a predictor for r=1. The fixed-point smoothers for the state and white noise are respectively computed by*

(17) $$\hat{x}(k-1|k-\alpha_{i}^{\left(k\right)})=\hat{x}(k-1|k-\alpha_{i-1}^{\left(k\right)})+K_{x}(k-1|k-\alpha_{i}^{\left(k\right)})\varepsilon (k-\alpha_{i}^{\left(k\right)})$$

(18) $$\hat{w}(k-1|k-\alpha_{i}^{\left(k\right)})=\hat{w}(k-1|k-\alpha_{i-1}^{\left(k\right)})+K_{w}(k-1|k-\alpha_{i}^{\left(k\right)})\varepsilon (k-\alpha_{i}^{\left(k\right)})$$

*where the innovation sequence $\varepsilon (k-\alpha_{i}^{\left(k\right)})$ and its covariance $Q_{\varepsilon }(k-\alpha_{i}^{\left(k\right)})$ are computed by*

(19) $$\varepsilon (k-\alpha_{i}^{\left(k\right)})=y(k-\alpha_{i}^{\left(k\right)})-\bar{\gamma }H\hat{x}(k-\alpha_{i}^{\left(k\right)}|k-\alpha_{i-1}^{\left(k\right)})$$

(20) $$Q_{\varepsilon }(k-\alpha_{i}^{\left(k\right)})={\bar{\gamma }}^{2}HP_{x}(k-\alpha_{i}^{\left(k\right)}|k-\alpha_{i-1}^{\left(k\right)})H^{T}+\bar{\gamma }(1-\bar{\gamma })Hq_{x}(k-\alpha_{i}^{\left(k\right)})H^{T}+Q_{v}$$

*The smoothing gain matrices $K_{x}(k-1|k-\alpha_{i}^{\left(k\right)})$ for the state and $K_{w}(k-1|k-\alpha_{i}^{\left(k\right)})$ for the white noise are computed by*

(21) $$K_{x}(k-1|k-\alpha_{i}^{\left(k\right)})=\bar{\gamma }[P_{x}(k-1|k-\alpha_{i-1}^{\left(k\right)})\Phi_{i}^{(k)T}+P_{xw}(k-1|k-\alpha_{i-1}^{\left(k\right)})\Gamma_{i}^{(k)T}]H^{T}Q_{\varepsilon }^{-1}(k-\alpha_{i}^{\left(k\right)})$$

(22) $$K_{w}(k-1|k-\alpha_{i}^{\left(k\right)})=\bar{\gamma }[P_{wx}(k-1|k-\alpha_{i-1}^{\left(k\right)})\Phi_{i}^{(k)T}+P_{w}(k-1|k-\alpha_{i-1}^{\left(k\right)})\Gamma_{i}^{(k)T}]H^{T}Q_{\varepsilon }^{-1}(k-\alpha_{i}^{\left(k\right)})$$

*The smoothing error covariance matrices $P_{x}(k-1|k-\alpha_{i}^{\left(k\right)})$ and $P_{w}(k-1|k-\alpha_{i}^{\left(k\right)})$, and the cross-covariance matrix $P_{xw}(k-1|k-\alpha_{i}^{\left(k\right)})$ for the state and white noise are computed by*

(23) $$P_{x}(k-1|k-\alpha_{i}^{\left(k\right)})=P_{x}(k-1|k-\alpha_{i-1}^{\left(k\right)})-K_{x}(k-1|k-\alpha_{i}^{\left(k\right)})Q_{\varepsilon }(k-\alpha_{i}^{\left(k\right)})K_{x}^{T}(k-1|k-\alpha_{i}^{\left(k\right)})$$

(24) $$P_{w}(k-1|k-\alpha_{i}^{\left(k\right)})=P_{w}(k-1|k-\alpha_{i-1}^{\left(k\right)})-K_{w}(k-1|k-\alpha_{i}^{\left(k\right)})Q_{\varepsilon }(k-\alpha_{i}^{\left(k\right)})K_{w}^{T}(k-1|k-\alpha_{i}^{\left(k\right)})$$

(25) $$P_{xw}(k-1|k-\alpha_{i}^{\left(k\right)})=P_{xw}(k-1|k-\alpha_{i-1}^{\left(k\right)})-K_{x}(k-1|k-\alpha_{i}^{\left(k\right)})Q_{\varepsilon }(k-\alpha_{i}^{\left(k\right)})K_{w}^{T}(k-1|k-\alpha_{i}^{\left(k\right)})$$

*The filtering (r=0) and prediction (r=1) error covariance matrices $P_{x}(k-\alpha_{i}^{\left(k\right)}|k-\alpha_{i-r}^{\left(k\right)})$ for the state are computed by*

(26) $$P_{x}(k-\alpha_{i}^{\left(k\right)}|k-\alpha_{i-r}^{\left(k\right)})=\Phi_{i}^{\left(k\right)}P_{x}(k-1|k-\alpha_{i-r}^{\left(k\right)})\Phi_{i}^{(k)T}+\Gamma_{i}^{\left(k\right)}P_{w}(k-1|k-\alpha_{i-r}^{\left(k\right)})\Gamma_{i}^{(k)T}\;+\Phi_{i}^{\left(k\right)}P_{xw}(k-1|k-\alpha_{i-r}^{\left(k\right)})\Gamma_{i}^{(k)T}+\Gamma_{i}^{\left(k\right)}P_{wx}(k-1|k-\alpha_{i-r}^{\left(k\right)})\Phi_{i}^{(k)T},\;r=0,1$$

The initial values are $P_{x}(k-1|k-\alpha_{0}^{\left(k\right)})=P_{x}(k-1|k-1)$, $P_{w}(k-1|k-\alpha_{0}^{\left(k\right)})=Q_{w}$, $P_{xw}(k-1|k-\alpha_{0}^{\left(k\right)})=0$, $\hat{x}(k-1|k-\alpha_{0}^{\left(k\right)})=\hat{x}(k-1|k-1)$ and $\hat{w}(k-1|k-\alpha_{0}^{\left(k\right)})=0$.

It is clear that we have the estimator at the state update point as $\hat{x}(k|k)=\hat{x}(k-\alpha_{N_{k}}^{\left(k\right)}|k-\alpha_{N_{k}}^{\left(k\right)})$ when $\alpha_{N_{k}}^{\left(k\right)}=0$ or $\hat{x}(k|k)=\hat{x}(k-\alpha_{N_{k+1}}^{\left(k\right)}|k-\alpha_{N_{k}}^{\left(k\right)})$ with $\alpha_{N_{k+1}}^{\left(k\right)}=0$, and $\beta_{N_{k+1}}^{\left(k\right)}=1/(1-\alpha_{N_{k}}^{\left(k\right)})$ when $\alpha_{N_{k}}^{\left(k\right)}\neq 0$.

**Proof.**  See Appendix A.

**Remark 2.**  In Theorem 2, if $N_{k}=1$ and $\alpha_{1}^{\left(k\right)}=0$, we have the filtering algorithm at the state update points for single-rate systems. If $N_{k}=0$, i.e., no samples in the interval (k−1,k], the predictor is used to estimate the state at the state update point k based on the estimator at the state update point k−1.

**Remark 3.**  It can be easily known that the proposed non-augmented recursive estimator has the computational order of magnitude $O(N_{k}p^{3})$ in the interval (k−1,k]. Compared with the augmented estimator [9] with the computational order of magnitude $O(N_{k}^{3}q^{3})$, our algorithm can obviously reduce the computational cost with the increase of the number $N_{k}$ of measurement samples in the interval (k−1,k] for a deterministic system with the fixed p and q. Meanwhile, it is more important that our filter can give the estimates not only at the state update points but also at the measurement sampling points within a state update period in real time. However, the estimator of [9] can only give the estimates at the state update points.

### 4.2. Computational Procedures of the Estimator

Based on Theorem 1 and Theorem 2, the computational procedures of our estimator at the state update points and at the measurement sampling points are addressed as follows:

Step 1. k=1, set the initial values $\hat{x}(0|0)=\mu_{0}$, $P_{x}(0|0)=P_{0}$, $P_{w}(0|0)=Q_{w}$, $P_{xw}(0|0)=0$ and $q_{x}(0)=\mu_{0}\mu_{0}^{T}+P_{0}$.

Step 2. Construct the model within a state update period (k−1,k] by Theorem 1.

Step 3. Compute the state estimators at the state update points and at the measurement sampling points:

(a) If $N_{k}\neq 0$ (i.e., there are samples within a state update period), obtain the state estimates $\hat{x}(k-\alpha_{i}^{\left(k\right)}|k-\alpha_{i}^{\left(k\right)})$ and the covariance matrices $P_{x}(k-\alpha_{i}^{\left(k\right)}|k-\alpha_{i}^{\left(k\right)})$ by Theorem 2.
(b) If $N_{k}=0$ (i.e., no sample within a state update period), obtain the estimate by prediction method in Remark 2, i.e., $\hat{x}(k|k)=\Phi \hat{x}(k-1|k-1)$, $P_{x}(k|k)=\Phi P_{x}(k-1|k-1)\Phi^{T}+\Gamma Q_{w}\Gamma^{T}$.

Step 4. k=k+1, set $P_{x}(k-1|k-\alpha_{0}^{\left(k\right)})=P_{x}(k-1|k-1)$, $P_{w}(k-1|k-\alpha_{0}^{\left(k\right)})=Q_{w}$, $P_{xw}(k-1|k-\alpha_{0}^{\left(k\right)})=0$, $\hat{x}(k-1|k-\alpha_{0}^{\left(k\right)})=\hat{x}(k-1|k-1)$ and $\hat{w}(k-1|k-\alpha_{0}^{\left(k\right)})=0$.

Go to Step 2.

## 5. Multi-Sensor Case

In the preceding section, we have presented a non-augmented optimal recursive estimator for single sensor system. In this section, we will discuss how to use the proposed algorithm to solve the multi-sensor case.

For a multi-sensor system, the state equation is the same as Equation (1) and the measurement equations are given as follows:

(27) $$y^{\left(l\right)}(k_{j}^{\left(l\right)})=\xi^{\left(l\right)}(k_{j}^{\left(l\right)})H^{\left(l\right)}x(k_{j}^{\left(l\right)})+v^{\left(l\right)}(k_{j}^{\left(l\right)})$$

where the superscript l denotes the lth sensor, L is the number of sensors, $y^{\left(l\right)}(k_{j}^{\left(l\right)})$ is the measurement at the sampling time $k_{j}^{\left(l\right)}$ for the lth sensor, $k_{j}^{\left(l\right)}$ is the sampling time of the *j*th measurement, $v^{\left(l\right)}(k_{j}^{\left(l\right)})$ is the measurement noise with zero mean and covariance$Q_{v^{\left(l\right)}}$, and $H^{\left(l\right)}$ is the measurement matrix. The variable $\xi^{\left(l\right)}(k_{j}^{\left(l\right)})$ is a Bernoulli distributed stochastic variable that takes values on 1 and 0 with the probability $\text{Prob}\{\xi^{\left(l\right)}(k_{j}^{\left(l\right)})=1\}={\bar{\gamma }}^{\left(l\right)}$, ${\bar{\gamma }}^{\left(l\right)}\in [0,1]$. We assume that $\xi^{\left(l\right)}(k_{j}^{\left(l\right)})$ is independent of w(k) and $v^{\left(l\right)}(k_{j}^{\left(l\right)})$ and x(0). Moreover $\xi^{\left(l\right)}(k_{j}^{\left(l\right)})$ satisfies that

(28) $$E\{\xi^{\left(l\right)}(k_{j}^{\left(l\right)}\}={\bar{\gamma }}^{\left(l\right)},\;E\{(\xi^{\left(l\right)}{\left(k_{j}^{\left(l\right)}-{\bar{\gamma }}^{\left(l\right)}\right)}^{2}\}={\bar{\gamma }}^{\left(l\right)}(1-{\bar{\gamma }}^{\left(l\right)}),\;E\{\xi^{\left(l\right)}(k_{j}^{l})-{\bar{\gamma }}^{\left(l\right)}\}=0,\;E\{\xi^{\left(l\right)}(k_{j}^{\left(l\right)})(1-\xi^{\left(l\right)}(k_{j}^{\left(l\right)}))\}=0,\;E\{\xi^{\left(l\right)}(k_{i}^{\left(l\right)})\xi^{\left(s\right)}(m_{j}^{\left(s\right)})\}={\bar{\gamma }}^{\left(l\right)}{\bar{\gamma }}^{\left(s\right)}(l\neq s\text{ or }k_{i}^{\left(l\right)}\neq m_{j}^{\left(s\right)})$$

We will adopt two types of methods to deal with the estimation problem of multi-sensor case: optimal fusion estimator and suboptimal fusion estimator.

### 5.1. Optimal Fusion Estimator

In fact, we can reorder the measurements $y^{\left(l\right)}(k_{j}^{\left(l\right)})$ of all sensors in each state update period (k−1,k] according to the order of sampling time $k_{j}^{\left(l\right)}$. If the sampling time of measurements from some sensors is same, i.e., sampling at the same time point, they will be combined to an augmented measurement at this sampling time. These reorder measurements can be considered from a certain single sensor. Then, our estimation algorithm in Section 4 can be applied to the optimal fusion estimation problem of multiple sensors.

When all sensors work healthily, the proposed optimal fusion algorithm can obtain the best accuracy. However, it has bad reliability, which means that a faulty sensor can lead to the optimal fusion estimator to diverge [26]. To improve the reliability, we give the following distributed suboptimal fusion algorithm.

### 5.2. Suboptimal Fusion Estimator

Firstly, apply our estimation algorithm in this paper to obtain the local estimators at the state update points based on the measurements from individual sensors, and then apply the covariance intersection (CI) fusion algorithm [27,28] to obtain the distributed suboptimal fusion estimator at the state update points. The reason that we adopt CI algorithm is that it avoids the computation of cross-covariance matrices between any two local estimators [26]. The CI fusion estimator can be given as follows [27,28]:

(29) $${\hat{x}}_{\text{CI}}(k|k)=\sum_{l=1}^{L}\omega^{\left(l\right)}(k)P_{\text{CI}}(k|k){\left[P_{x}^{\left(l\right)}(k|k)\right]}^{-1}{\hat{x}}^{\left(l\right)}(k|k)$$

(30) $$P_{\text{CI}}(k|k)={\left[\sum_{l=1}^{L}\omega^{\left(l\right)}(k){\left[P_{x}^{\left(l\right)}(k|k)\right]}^{-1}\right]}^{-1}$$

where $P_{\text{CI}}(k|k)$ is the upper bound of variance of the CI fusion estimator. The optimal weighted coefficients $\omega^{\left(l\right)}(k)$ satisfy $0\leq \omega^{\left(l\right)}(k)\leq 1$ and $\sum_{l=1}^{L}\omega^{\left(l\right)}(k)=1$, and can be obtained by solving the following optimization problem:

(31) $$\underset{\sum_{l=1}^{L}\omega^{\left(l\right)}(k)=1,0\leq \omega^{\left(l\right)}(k)\leq 1}{\min}\text{tr}(P_{CI}(k|k))$$

The nonlinear optimization problem Equation (29) does not generally have the analytical solutions. It can be solved by numerical methods, which can be carried out by the function “fmincon” in Matlab optimization toolbox. It only involves the scalar optimization problem.

The proposed distributed CI fusion estimator has the advantage of good robustness and flexibility, i.e., good reliability since it has the distributed parallel structure that is convenient for detection and isolation of a faulty sensor. Certainly, when all sensors work healthily, it has worse accuracy than the optimal fusion estimator. However, it has better accuracy than local estimators [27,28].

**Remark 4.**  The proposed optimal fusion algorithm is a non-augmented method. Certainly, we can also use the centralized fusion way [9] that combines the measurements from all sensors within a state update period into an augmented measurement and then use standard Kalman filter to solve. However, it is well known that this way has the expensive computational burden with the increase of the number of sensors [26].

## 6. Simulation Research

In this section, a spring-mass system that has been widely adopted in many studies [29,30,31] will be used to verify the effectiveness of our algorithms:

(32) $$\dot{x}(t)=\left[\begin{array}{cccc} 0 & 0 & 1 & 0\\ 0 & 0 & 0 & 1\\ -\frac{k_{1}+k_{2}}{M_{1}} & \frac{k_{2}}{M_{1}} & -\frac{\mu }{M_{1}} & 0\\ \frac{k_{2}}{M_{2}} & -\frac{k_{2}}{M_{2}} & 0 & -\frac{\mu }{M_{2}} \end{array}\right]x(t)+\left[\begin{array}{c} 0\\ 0\\ \frac{1}{M_{1}}\\ \frac{1}{M_{2}} \end{array}\right]w(t)$$

where $x(t)={\left[\begin{array}{cccc} x_{1}(t) & x_{2}(t) & {\dot{x}}_{1}(t) & {\dot{x}}_{2}(t) \end{array}\right]}^{T}$, $x_{1}$ and $x_{2}$ are the positions of mass $M_{1}$ and mass $M_{2}$, respectively; $k_{1}$ and $k_{1}$ are the spring constants of spring 1 and spring 2, respectively; and μ denotes the viscous friction coefficient between the mass blocks and the horizontal surface. Moreover, the measurement equations are given as Equation (27) with L = 3. $v^{\left(i\right)}(k_{j}^{\left(i\right)})$, i=1,2,3 are independent Gaussian noises with zero means and variances $Q_{v^{\left(i\right)}}$ and uncorrelated with the process noise w(k) with zero-mean and variance $Q_{w}$. We set $Q_{w}=2$, $Q_{v^{\left(1\right)}}=2$, $Q_{v^{\left(2\right)}}=1$, $Q_{v^{\left(3\right)}}=3$, $H^{\left(1\right)}=\left[\begin{array}{cccc} 1 & 1 & 1 & 0 \end{array}\right]$, $H^{\left(2\right)}=\left[\begin{array}{cccc} 0 & 1 & 0 & 0 \end{array}\right]$, $H^{\left(3\right)}=\left[\begin{array}{cccc} 0 & 0 & 1 & 1 \end{array}\right]$, ${\bar{\gamma }}^{\left(1\right)}=0.7$, ${\bar{\gamma }}^{\left(2\right)}=0.9$, ${\bar{\gamma }}^{\left(3\right)}=0.8$, $M_{1}=1$, $M_{2}=0.5$, $k_{1}=1$, $k_{2}=1$, μ=0.5, and the sampling period T=0.1 s, we obtain the following system parameter matrices by discretization:

(33) $$\Phi =\left[\begin{array}{cccc} 0.9902 & 0.0049 & 0.0972 & 0.0002\\ 0.0096 & 0.9903 & 0.0003 & 0.0948\\ -0.1941 & 0.0969 & 0.9416 & 0.0047\\ 0.1891 & -0.1894 & 0.0095 & 0.8955 \end{array}\right],\;\Gamma =\left[\begin{array}{c} 0.0049\\ 0.0097\\ 0.0975\\ 0.1900 \end{array}\right]$$

We set the initials as x(0)=0 and $P_{0}=0.1I_{4}$. We take 100 sampling data.

**Figure 2:**
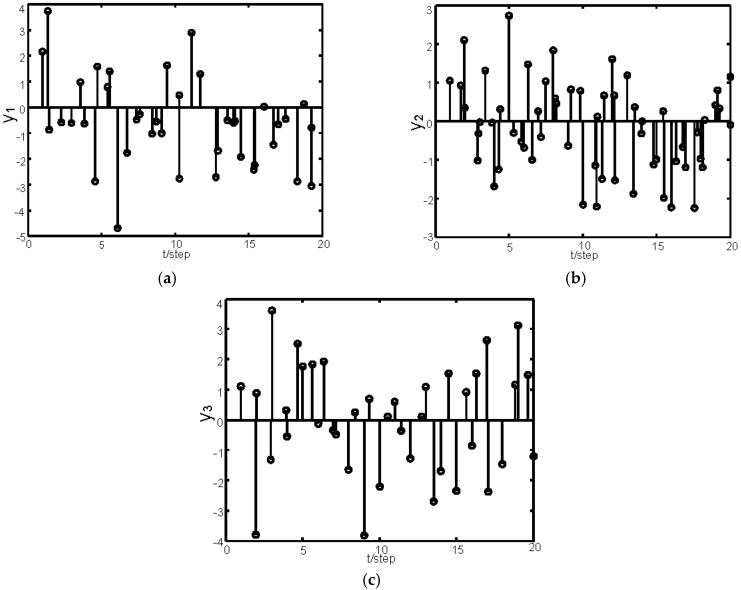
Samples of sensors 1–3 in [1,20]: (**a**) samples of the first sensor; (**b**) samples of the second sensor; and (**c**) samples of the third sensor.

**Figure 3:**
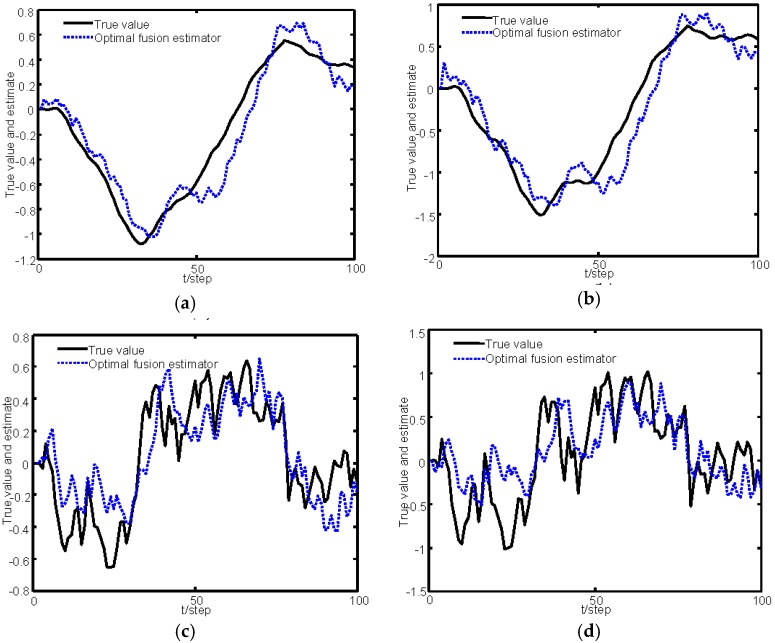
Optimal fusion estimator: (**a**) tracking for the position of *M*_1_; (**b**) tracking for the position of *M*_2_; (**c**) tracking for the velocity of *M*_1_; and (**d**) tracking for the velocity of *M*_2_.

**Figure 4:**
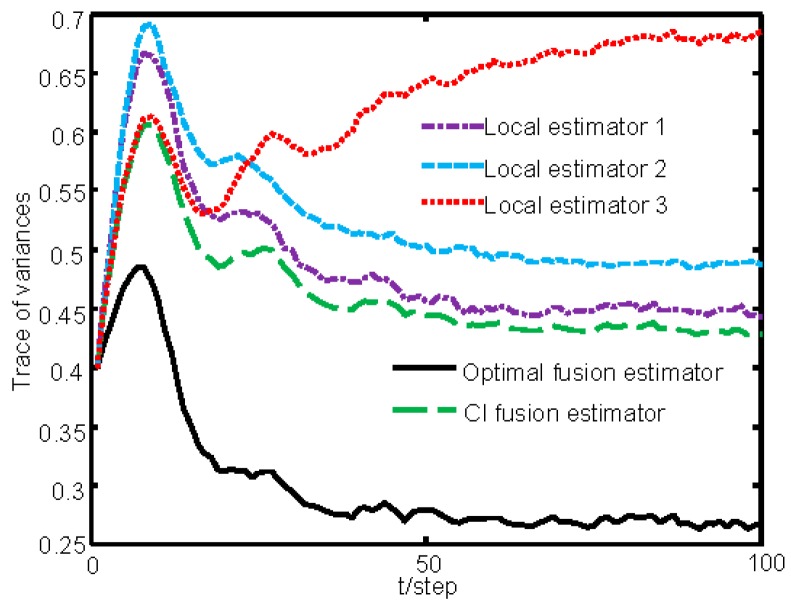
Comparison of the traces of variances of local estimators and fusion estimators.

Figure 2 gives the sketch map of the sampling data of the three sensors within the time interval 0–20. Figure 3 gives the tracking performance of the optimal fusion estimator, where the solid curves denote the true values and the dashed ones denote the estimators. Figure 4 gives the comparisons curves of the traces of variances for local estimators and the fusion estimators. From Figure 4, we can see that our optimal fusion estimator has best accuracy and CI fusion estimator has better accuracy than local estimators.

Then Monte Carlo simulation is carried out to compare the algorithms in our paper and [9]. The mean square errors (MSEs): ${\text{MSE}}_{i,s}(k)=\frac{1}{N}\sum_{j=1}^{N}{\left(x_{i}^{j}(k)-{\hat{x}}_{i,s}^{j}(k|k)\right)}^{2}$, *i*=1, 2, 3, 4; *s*=our paper, [9]) will be used to evaluate the estimation performance of the two estimation algorithms, where the subscript *i* denotes the *i*th components and *s* denotes our filter or that in [9], respectively, and N is the number of Monte Carlo runs.

**Figure 5:**
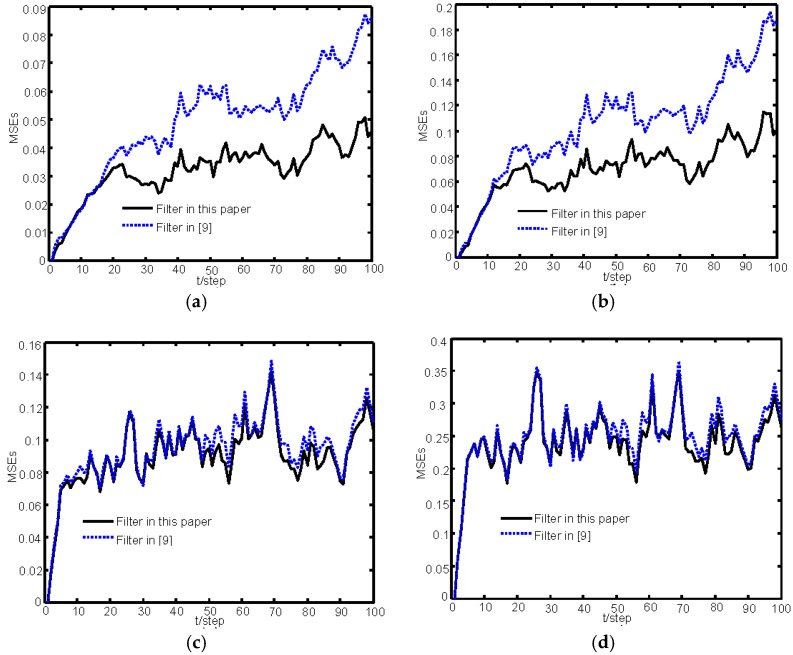
Comparison of MSEs of the estimators in this paper and [9] for sensor 1: (**a**) MSEs of the position estimators of *M*_1_; (**b**) MSEs of the position estimators of *M*_2_; (**c**) MSEs of the velocity estimators of *M*_1_; and (**d**) MSEs of the velocity estimators of *M*_2_.

In Figure 5, the sensor 1 is randomly employed to compare the algorithms in our paper and [9]. The comparison of MSEs simulated by 100 Monte Carlo runs is shown in Figure 5. Because Yan, L. et al. [9] does not consider the effect of missing measurements, from Figure 5, we can see that the proposed algorithm has better accuracy than the one in [9] when the missing measurements occur in sensors.

**Figure 6:**
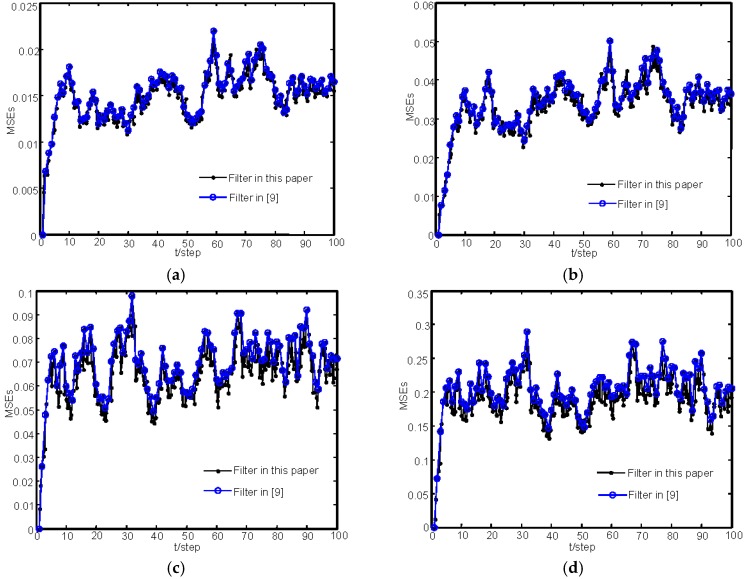
Comparison of MSEs of the estimators in this paper and [9] for sensor 1 without missing measurements: (**a**) MSEs of the position estimators of *M*_1_; (**b**) MSEs of the position estimators of *M*_2_; (**c**) MSEs of the velocity estimators of *M*_1_; and (**d**) MSEs of the velocity estimators of *M*_2_.

Next, we will make the comparison with [9] when there are no missing measurements. The sensor 1 is employed with ${\bar{\gamma }}^{\left(1\right)}=1$ in simulation. Figure 6 gives the comparison of MSEs simulated by 100 Monte Carlo runs for the estimators in this paper and [9]. In Figure 6, the estimates at the state update points and the measurement sampling points within a state update period are all shown by using the our proposed algorithm. Moreover, from Figure 6, we can see that the proposed algorithm has the same accuracy as the augmented one in [9] at the state update points when there are no missing measurements. It is also significant from Remark 3 that our estimator has smaller computational burden than [9]. To compare the computation time between the two algorithms, we use the “unifrnd” function in Matlab to sample 2, 3, 4, and 5 measurements within a state update period, respectively, and give the average of 50 run time of the two algorithms in Table 1. The used computer is Lenovo with the CPU speed of 3.20 GHz (Intel (R) Core (TM) i5-3470 CPU) and the used Matlab version is Matlab R2012a. From Table 1, we can see that our proposed algorithm can save the run time compared with that in [9] with the increase of the number of sampling points within a state update period.

## 7. Conclusions

A non-augmented recursive estimator has been designed for a class of non-uniform sampling systems with missing measurements, where the system state is updated uniformly and the measurements are sampled randomly. A state space model is constructed to depict the dynamics at the measurement sampling points within a state update period. The proposed estimator dependent on the missing rate can provide the state estimates not only at the state update points but also at the measurement sampling points within a state update period. Compared with [9], our algorithm has better accuracy when there are missing measurements and the same accuracy at the state update points when there are no missing measurements. For multi-sensor systems with missing measurements, our algorithm can also deal with the optimal fusion estimation by reordering the measurements from all sensors. A distributed suboptimal fusion estimator is also given using the covariance intersection (CI) fusion algorithm.

## Figures and Tables

**Table 1:** Average time of 50 runs.

Number of Samples	Our Algorithm	[9]
2	0.062064 s	0.066064 s
3	0.078872 s	0.086958 s
4	0.092339 s	0.112633 s
5	0.103354 s	0.146654 s

## Appendix A. The Proof of Theorem 2

By using the projection theory [32], we readily have Equations (16)–(19), where the filtering gain matrices for the state and input white noise are, respectively, defined as

(A1) $$K_{x}(k-1|k-\alpha_{i}^{\left(k\right)})=E\left\{x(k-1)\varepsilon^{T}(k-\alpha_{i}^{\left(k\right)})\right\}Q_{\varepsilon }^{-1}(k-\alpha_{i}^{\left(k\right)})$$

(A2) $$K_{w}(k-1|k-\alpha_{i}^{\left(k\right)})=E\left\{w(k-1)\varepsilon^{T}(k-\alpha_{i}^{\left(k\right)})\right\}Q_{\varepsilon }^{-1}(k-\alpha_{i}^{\left(k\right)})$$

Substituting Equation (10) into Equation (19), the innovation $\varepsilon (k-\alpha_{i}^{\left(k\right)})$ can be rewritten as

(A3) $$\varepsilon (k-\alpha_{i}^{\left(k\right)})=(\xi (k-\alpha_{i}^{\left(k\right)})-\bar{\gamma })Hx(k-\alpha_{i}^{\left(k\right)})+\bar{\gamma }H\tilde{x}(k-\alpha_{i}^{\left(k\right)}|k-\alpha_{i-1}^{\left(k\right)})+v(k-\alpha_{i}^{\left(k\right)})$$

where the prediction error $\tilde{x}(k-\alpha_{i}^{\left(k\right)}|k-\alpha_{i-1}^{\left(k\right)})=x(k-\alpha_{i}^{\left(k\right)})-\hat{x}(k-\alpha_{i}^{\left(k\right)}|k-\alpha_{i-1}^{\left(k\right)})$. Substituting Equation (A3) into the innovation sequence covariance $Q_{\varepsilon }(k-\alpha_{i}^{\left(k\right)})=E\left\{\varepsilon (k-\alpha_{i}^{\left(k\right)})\varepsilon^{T}(k-\alpha_{i}^{\left(k\right)})\right\}$ leads to Equation (20).

Subtracting Equation (16) with r=1 from Equation (9) yields the prediction error equation

(A4) $$\tilde{x}(k-\alpha_{i}^{\left(k\right)}|k-\alpha_{i-1}^{\left(k\right)})=\Phi_{i}^{\left(k\right)}\tilde{x}(k-1|k-\alpha_{i-1}^{\left(k\right)})+\Gamma_{i}^{\left(k\right)}\tilde{w}(k-1|k-\alpha_{i-1}^{\left(k\right)})$$

where the smoothing errors $\tilde{x}(k-1|k-\alpha_{i-1}^{\left(k\right)})=x(k-1)-\hat{x}(k-1|k-\alpha_{i-1}^{\left(k\right)})$ and $\tilde{w}(k-1|k-\alpha_{i-1}^{\left(k\right)})=$ w(k−1)− $\hat{w}(k-1|k-\alpha_{i-1}^{\left(k\right)})$. Using Equations (A3) and (A4), we have

(A5) $$E\left\{x(k-1)\varepsilon^{T}(k-\alpha_{i}^{\left(k\right)})\right\}=\bar{\gamma }E\{x(k-1)(\Phi_{i}^{\left(k\right)}\tilde{x}(k-1|k-\alpha_{i-1}^{\left(k\right)}){+\Gamma_{i}^{\left(k\right)}\tilde{w}(k-1|k-\alpha_{i-1}^{\left(k\right)}))}^{T}\}H^{T}\;=\bar{\gamma }[P_{x}(k-1|k-\alpha_{i-1}^{\left(k\right)})\Phi_{i}^{(k)T}+P_{xw}(k-1|k-\alpha_{i-1}^{\left(k\right)})\Gamma_{i}^{(k)T}]H^{T}$$

where $x(k-1)=\hat{x}(k-1|k-\alpha_{i-1}^{\left(k\right)})+\tilde{x}(k-1|k-\alpha_{i-1}^{\left(k\right)})$, $\hat{x}(k-1|k-\alpha_{i-1}^{\left(k\right)})⊥\tilde{x}(k-1|k-\alpha_{i-1}^{\left(k\right)})$ and $\hat{x}(k-1|k-\alpha_{i-1}^{\left(k\right)})⊥$ $\tilde{w}(k-1|k-\alpha_{i-1}^{\left(k\right)})$ have been used, where the symbol ⊥ denotes orthogonality. Substituting Equation (A5) into Equation (A1) yields Equation (21). Similarly, we prove Equation (22) holds.

Subtracting Equation (17) from x(k−1) yields the smoothing error equation

(A6) $$\tilde{x}(k-1|k-\alpha_{i}^{\left(k\right)})=\tilde{x}(k-1|k-\alpha_{i-1}^{\left(k\right)})-K_{x}(k-1|k-\alpha_{i}^{\left(k\right)})\varepsilon (k-\alpha_{i}^{\left(k\right)})$$

The smoothing error covariance matrix is derived as follows:

(A7) $$P_{x}(k-1|k-\alpha_{i}^{\left(k\right)})=E\left\{\tilde{x}(k-1|k-\alpha_{i}^{\left(k\right)}){\tilde{x}}^{T}(k-1|k-\alpha_{i}^{\left(k\right)})\right\}\;=P_{x}(k-1|k-\alpha_{i-1}^{\left(k\right)})+K_{x}(k-1|k-\alpha_{i}^{\left(k\right)})Q_{\varepsilon }(k-\alpha_{i}^{\left(k\right)})K_{x}^{T}(k-1|k-\alpha_{i}^{\left(k\right)})\;-E\left\{\tilde{x}(k-1|k-\alpha_{i-1}^{\left(k\right)})\varepsilon^{T}(k-\alpha_{i}^{\left(k\right)})\right\}K_{x}^{T}(k-1|k-\alpha_{i}^{\left(k\right)})\;-K_{x}(k-1|k-\alpha_{i}^{\left(k\right)})E\left\{\varepsilon (k-\alpha_{i}^{\left(k\right)}){\tilde{x}}^{T}(k-1|k-\alpha_{i-1}^{\left(k\right)})\right\}$$

Using Equation (A1) and note that

(A8) $$E\left\{\tilde{x}(k-1|k-\alpha_{i-1}^{\left(k\right)})\varepsilon^{T}(k-\alpha_{i}^{\left(k\right)})\right\}=E\left\{x(k-1)\varepsilon^{T}(k-\alpha_{i}^{\left(k\right)})\right\}\;=K_{x}(k-1|k-\alpha_{i}^{\left(k\right)})Q_{\varepsilon }(k-\alpha_{i}^{\left(k\right)})$$

Substituting Equation (A8) into Equation (A7) leads to Equation (23). Similarly, we can prove Equations (24) and (25) hold.

Subtracting Equation (16) with r=0 from Equation (9) yields the filtering error equation

(A9) $$\tilde{x}(k-\alpha_{i}^{\left(k\right)}|k-\alpha_{i}^{\left(k\right)})=\Phi_{i}^{\left(k\right)}\tilde{x}(k-1|k-\alpha_{i}^{\left(k\right)})+\Gamma_{i}^{\left(k\right)}\tilde{w}(k-1|k-\alpha_{i}^{\left(k\right)})$$

Substituting Equations (A4) and (A9) into $P_{x}(k-\alpha_{i}^{\left(k\right)}|k-\alpha_{i-r}^{\left(k\right)})=E\{\tilde{x}(k-\alpha_{i}^{\left(k\right)}|k-\alpha_{i-r}^{\left(k\right)})$ ${\tilde{x}}^{T}(k-\alpha_{i}^{\left(k\right)}|k-\alpha_{i-r}^{\left(k\right)})\}$, r=0,1 leads to Equation (26). This proof is completed.
